# Research on intervention strategies for fire rescue personnel’s competency frustration: EEG experimental validation

**DOI:** 10.3389/fpsyg.2024.1455117

**Published:** 2024-11-13

**Authors:** Yarong Wang, Runyu Zhang, Ying Liu, Jie Ren, Guosheng Zhang

**Affiliations:** ^1^Department of Economics and Management, Inner Mongolia University of Science and Technology, Baotou, China; ^2^Industrial Informatization and Industrial Innovation Research Centre, Inner Mongolia University of Science and Technology, Baotou, China; ^3^Firefighting and Rescue Command of Baotou Fire and Rescue Detachment, Baotou, China

**Keywords:** competence frustration, fire rescue personnel, frontal alpha asymmetry (FAA), EEG, cognitive science experiments

## Abstract

In order to accurately measure the level of competency frustration and what measures to take to alleviate the negative effects of competency frustration, 35 graduate students were selected to verify the effect of the frontal lobe *α* asymmetry (FAA) as a judgement of the competence frustration level using EEG experimental method. On this basis, through two stopwatch stopping experiments, 108 fire rescue personnel were selected to conduct the experiments to investigate the intervention effects of developmental feedback and compassion-focused therapy in turn. The results showed that frontal *α* asymmetry could be used as an EEG indicator for judging competency frustration, and the intervention method of compassion-focused therapy reduced the level of competency frustration of the subjects, while developmental feedback interventions did the opposite. The difference in the effects of the two intervention methods indicates that when intervening in competence frustration, it is easier to reduce the competency frustration by focusing on the subjects themselves than focusing on the completion of the task.

## 1 Introduction

Creating a favorable safety environment is an essential emergency mission for firefighters and rescue personnel to comprehensively enhance the level of disaster prevention, reduction, and relief, and to advance the construction of a strong nation and national rejuvenation. When the people are in dire need, they charge forward to rescue them from fire and water and save them from peril. Due to the particularity of firefighting and rescue work, i.e., exposing to extreme working environment, high-intensity workload pressure, and potential life risks, it may have adverse effects on their physical and mental health leading to anxiety stemming from feelings of inadequacy in meeting job demands. Competency need is considered as a basic, innate psychological need of the individual, and competence frustration refers to the individual’s perceived lack of competence and feelings of some kind of failure in the task or work, etc. (Bartholomew et al., 2014). Research has shown that job stress, emotional exhaustion, negative feedback, and demanding management are all important triggers for competence need frustration (Deci et al., 2017; Vansteenkiste et al., 2020; Trepanier et al., 2016). The creation and spread of competency need frustration can have numerous negative consequences for individuals and organizations. Competency frustration-induced health problems have become a significant factor in the combat effectiveness of the fire brigades. (Ma et al., 2007). For fire and rescue personnel, competency need frustration can lead to negative emotions (Bartholomew et al., 2011; Gunnell et al., 2013; Unanue et al., 2017), depressive symptom (Van den Broeck et al., 2014), unhealthy state (Chen et al., 2015) and unhappiness (Gillet et al., 2012). For the firefighting brigade, competency frustration reduces firefighters’ autonomous motivation and will to fight, thereby negatively affecting the development and combat effectiveness of the firefighting brigade. Therefore, it is necessary to study the intervention methods and explore the effective strategies to eliminate the competence need frustration of firefighters and rescue personnel.

### 1.1 Measurement of competence needs frustration

Established research has primarily used scales to assess the measurement of competency need frustration. For example, Bartholomew et al. (2014) developed a basic psychological frustration scale for American athletes, consisting of three main dimensions: autonomy need frustration, competence need frustration, and belonging need frustration; Chen (2015) developed the Basic Psychological Needs Satisfaction and Basic Psychological Frustration Scales in the Work Field for corporate employees; Chen et al. (2020) developed the Basic Psychological Frustration Scale for Teachers based on the Chinese educational context, and all the above scales have been confirmed to have good reliability and validity. However, as an intrinsic psychological response, the traditional measures (e.g., questionnaires or free-choice methods) have shortcomings such as reliance on retrospectives, subjective biases, and have large errors. With the rapid development of cognitive neuroscience, brain science and other technologies, we can now use cognitive neuroscience techniques to monitor the cognitive processing activities of the brain in real time (Liu and Mai, 2021), uncovering the “black box” of the neural level of mental activity to provide a more accurate and objective measurement of the level of individual’s frustration of the need for competence. At present, no scholars at home and abroad have proposed specific EEG indicators for the psychological condition of competence frustration.

Frontal Alpha Asymmetry (FAA) refers to the difference between left and right brain alpha wave activity in the frontal regions of the brain during EEG recordings. After decades of developmental research, frontal alpha asymmetry (FAA) has been recognized as a biomarker of depression and anxiety and has been evaluated in many studies (Gao et al., 2021). Liu et al. (2022) compared the differences in FAA between patients with depression and healthy control subjects and found that the right frontal lobe of the depressed patients had a stronger intensity of activity than that of the left frontal lobe, and their frontal asymmetry showed a pattern opposite to that of healthy individuals. This opposite pattern is believed to be one of the potential neurophysiological hallmarks of the patients with depression. Meanwhile, FAA has also been used as an indicator to measure anxiety (Feldmann et al., 2018), subjective well-being (Wutzl et al., 2023), negative emotions (Barros et al., 2022), negative self-perceptions (Millis et al., 2022), and has achieved corresponding research results. Considering that individuals suffering from competence need frustration often experience negative emotions of depression, anxiety, and outward manifestations of depression, this paper attempts to use FAA as an electroencephalographic indicator for measuring competence need frustration. Some studies have also found that changing the frontal alpha wave asymmetry index through neurofeedback methods can improve depressive symptoms, which is of great significance for the diagnosis and treatment of depression (Mennella et al., 2017; Zotev et al., 2020). This also suggests that FAA indicators can not only be used to assess the level of competence need frustration, but also help to assess the intervention effect of competence need frustration. Based on the above research bases, this paper chooses frontal alpha asymmetry as an EEG indicator to measure the level of competence need frustration and to judge the effect of high or low level of competence need frustration.

### 1.2 Interventions for competence needs thwarting

Currently, the research on competency need frustration focuses on two aspects: first, it is committed to accurately measuring the level of competency need frustration, and has developed the Competency Need Frustration Scale, which has been modified according to the actual situation; second, it is to explore the antecedent and consequent variables of competency need frustration. Few scholars have examined intervention measures after individuals suffer from competency need frustration; therefore, the research on competency need frustration lacks the final link. This study will explore the intervention strategies for competency need frustration in terms of both organizational-level factors (developmental feedback) and individual-level factors (empathy-focused therapy).

Developmental feedback refers to the directional provision of useful or valuable information that enables people to learn, develop and improve in their work (Zhou, 2003). Developmental feedback emphasizes the concern and expectation for employees’ future career development, enhances employees’ beliefs, and improves individuals’ psychological resilience and recovery (Yao et al., 2014). At the same time, developmental feedback can make employees feel encouraged and supported, and believe that they have been respected and cared for, which is conducive to promoting the quality of interpersonal relationships and enhancing psychological resilience (Meng et al., 2024; Yin and Zheng, 2011). Developmental feedback, on the one hand, can stimulate employees to generate intrinsic motivation such as self-innovation expectations (Song and Wang, 2015), innovation capabilities (Weilin and Xinqi, 2018), show higher work dedication (Eva et al., 2019), and promote the quality of interpersonal interactions (Sommer and Kulkarni, 2012), which in turn effectively reduces employees’ s emotional exhaustion (Liao and Wu, 2020); on the other hand, developmental feedback, as positive informational feedback, can promote employees’ work skill improvement and competence, thereby gaining satisfaction and a sense of accomplishment (Li et al., 2022). Both of these aspects have a significant effect on the individual’s level of competency need frustration.

Compassion-focused therapy (CFT) trains individuals to feel compassion by empathizing with their own past and current feelings toward people or events, and to focus external sympathy on themselves from different perspectives. This training method can effectively train self-compassion while reducing negative emotions such as depression, anxiety, and shame (Gilbert and Procter, 2006). Scholars at home and abroad have utilized the technique of empathy-focused therapy to treat a variety of psychological problems. Studies have shown that empathy-focused therapy has a good effect in effectively reducing a series of psychological problems and symptoms such as depression, anxiety, shame, and self-criticism in patients with post-traumatic stress disorder and eating disorders. For example, Bowyer et al. (2014) conducted compassion-focused therapy intervention on 32 adolescents who had experienced early interpersonal trauma and found that their depression, shame, and self-harm levels were significantly reduced. Vrabel et al. (2019) conducted a randomly- controlled trial on 144 subjects who had experienced early trauma and found that compassion-focused therapy can significantly reduce subjects’ post-traumatic related symptoms and improve their life satisfaction. Compassion-focused therapy is a relatively novel form of psychotherapy, and in recent years, more and more scholars have begun to pay attention to compassion-focused therapy and conduct experimental studies on a variety of populations, proving the feasibility, acceptability, and efficacy of compassion-focused therapy in alleviating the symptoms associated with a range of problems and conditions such as depression, anxiety, shame, and self-criticism (Sommers et al., 2018).

Based on this, this paper selects the frontal alpha asymmetry EEG index to measure the level of competence need frustration, and designs Experiment 1: to verify the effect of frontal alpha asymmetry as a judgment of the level of competence need frustration. Design of Experiment 2: to verify the effects and differences of the two intervention measures of developmental feedback and empathy-focused therapy on competence need frustration and explain their neural mechanisms. The study proposes to address two problems: the first is to investigate the experimental EEG measure of competency need frustration in fire and rescue personnel; the second is to investigate effective interventions for fire rescue personnel after they have suffered from competency need frustration.

## 2 Theory construction

Scholars have been increasingly concerned about the phenomenon of individuals suffering from competence need frustration. They have carried out preliminary discussions on the measurement, causes and negative outcomes of competence need frustration, and have achieved a series of results, but there are still some deficiencies in the research. Firstly, as a psychological feeling or emotion, competence frustration is mainly measured by free-choice and self-report methods, which have certain deviations. With the cognitive neuroscience technology, real-time monitoring of cognitive processing activities in the brain can be realized, so that the level of competency frustration suffered by individuals can be measured accurately and objectively. Secondly, the research subjects of scholars on competence frustration are mainly teenagers, athletes and corporate employees, and few people in other industries are involved. This study attempts to take firefighters and rescue personnel as the research subjects. Third, the existing studies mainly focus on the antecedents and consequences of competence need frustration, and rarely explore the intervention strategies after individuals suffer from competence need frustration.

**Figure 1:**
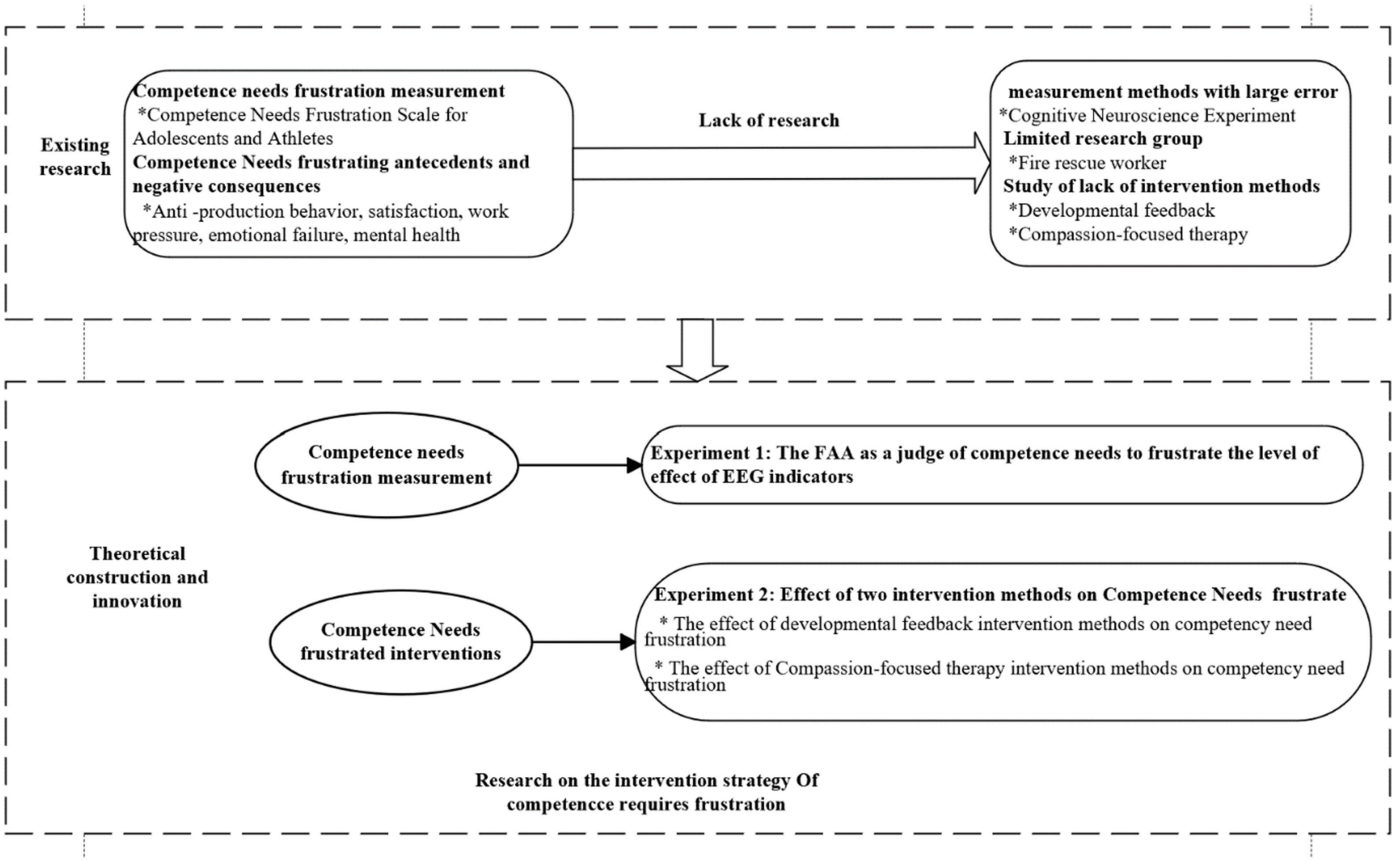
Theoretical construction of this research.

This study attempts to select firefighters and rescue personnel as the research subjects from the perspective of cognitive neuroscience, measure the level of competence need frustration using EEG technology, and seek to guide firefighters and rescue personnel to get rid of the intervention strategies of competence need frustration, so as to maintain and improve their competence. Based on this, the theoretical construction of the study is shown in Figure 1.

## 3 Research methods

### 3.1 Competence frustration induction experiments

#### 3.1.1 Subjects and experimental materials

The G*Power tool was used in this study to calculate the required sample size for the study (Faul et al., 2009), and the ideal statistical test power and effect size were both higher than 0.8. Using this as a criterion, the total sample required in a one-way, two-level within-groups design was 15 subjects (significance level *α* = 0.05, test validity 1-*β* = 80%). Thirty-five current graduate students were recruited to participate in this experiment to meet the experimental sample size requirement, and all subjects met the requirements of right-handedness, normal vision or corrected vision, no history of neurology or mental illness, and were between the ages of 23 and 27 years old, with a mean age of 25.01 years old (SD = 1.27 years old). Informed consent was obtained from all subjects.

Drawing on the experimental methods used by scholars to induce frustration of the need for competence, a stopwatch task was proposed as the experimental material (Meng and Ma, 2015; Murayama et al., 2010). In the stopwatch task, subjects were required to estimate the duration of 3 s as accurately as possible without the help of external tools. Specifically, a stopwatch icon appeared in the center of the screen to indicate the start of the task. If subjects felt that 3 s had elapsed since the start of the task, they should confirm it by clicking the mouse immediately. If the stopwatch stopped within the success interval, it was regarded as a success, and the exact time of the keystroke would be fed back to the subject through the green box and green font; if the stopwatch stopped outside the success interval, it was regarded as a failure, and the exact time of the keystroke would be fed back to the subject through the red box and red font. The experiment lasted for a total of forty rounds, after which the screen would display the number of successes and the success rate of the subjects. In addition, in order to validate the experiment, the Competency Needs Frustration Scale developed by Fang et al. (2018) was used, which consists of a four-question likert-7 scale for scoring, which can be selected from 1 (Strongly Disapprove) to 7 (Strongly Approve). The higher the score, the higher the degree of their competency need frustration.

#### 3.1.2 Experimental procedures

**Figure 2:**
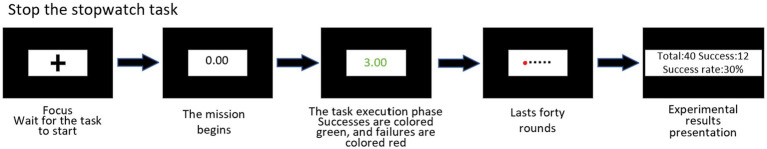
Flow chart of stopwatch stopping time experiment.

Prior to the start of the experiment, after the subjects arrived at the EEG laboratory, the experimenter introduced the precautions for the EEG experiment and the subjects signed an informed consent form. Subsequently, the subjects were given the EEG equipment and prepared to start the experiment. The experiment was divided into two stages. The first stage required the subjects to take a two-minute rest with their eyes closed to calm down, and EEG data were collected in the resting state. In the second stage, the subjects were required to complete the stopwatch task according to the on-screen instructions. At the beginning of each round of the task, a “+” will appear in the center of the screen, with a duration of 800-1200 ms randomly, reminding the subjects to focus their attention, followed by the stopwatch task starting from 0 s. The subject’s goal was to try their best to stop the stopwatch at around 3 s. Drawing on the reference settings of previous scholars, the success interval was set to [2.93 s ~ 3.07 s] (Murayama et al., 2015), and the experiment lasted for 40 rounds. The flow chart of the experiment is shown in Figure 2. At the end of the experiment, the researchers asked the subjects to fill out a questionnaire on “Competence Needs Frustration” to validate the analysis.

#### 3.1.3 Data collection

In this study, EEG data were collected using an Ergo LAB EEG wearable electroencephalogram with a sampling rate of 500 Hz. Each subject wore an Ergo LAB 32-conductivity electrode cap, and the cap electrodes were based on the International 10–20 System. The default electrode was used as the reference electrode for EEG data collection, and the resistance on each electrode was controlled not to exceed 5 k *Ω* to ensure the data quality. During the EEG experiments, the Ergo LAB human-computer environment synchronization platform was used to collect EEG data from the subjects throughout the process.

#### 3.1.4 Data processing

**Figure 3:**
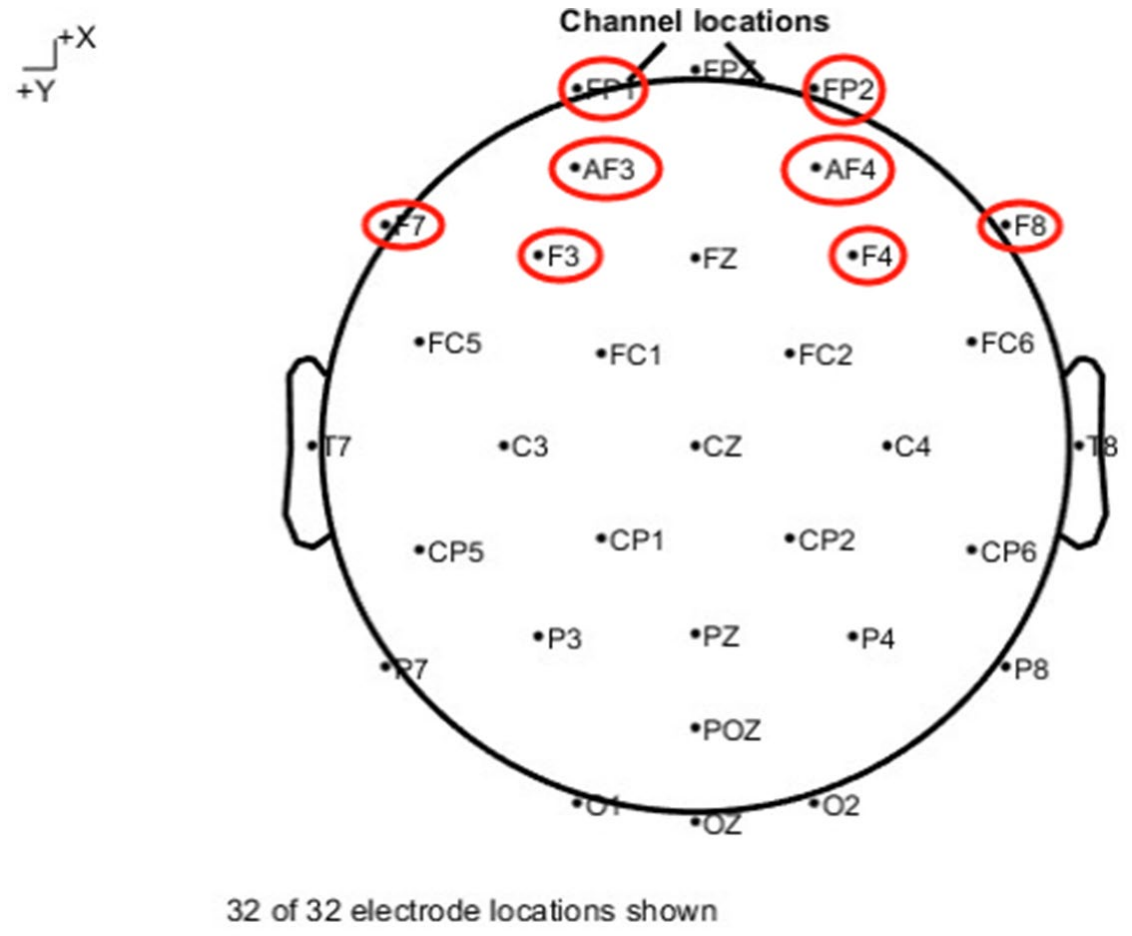
Distribution of 32 electrode sites in the brain and selected electrode sites.

The quality of the acquired EEG data was checked, and the subject data with large artifacts were removed. The EEG data were pre-processed, the CZ point was set as the reference electrode, and the T7 and T8 points were selected as the reference values for the re-referencing method for the bilateral mastoid process; the re-referenced data were band-pass filtered from 0.1 to 30 Hz, and the data were segmented in a segment of 1,000 data points (1 s). The artifacts such as ophthalmoscopes and electromyograms were removed using the independent component analysis method. The pre-processed EEG data were divided into two segments, the first segment was the resting EEG data, i.e., the EEG data of the subjects when they were resting with eyes closed; the second segment was the EEG data of the subjects when they completed the stopwatch experiment. Then, the power spectral density value was used to assess the intensity of frontal lobe activity. The band frequency of *α*-wave selected in this paper was 8 ~ 13hz, and the electrode points were mainly selected from the prefrontal lobe region of FP1, FP2, AF3, AF4, F3, F4, F7, and F8, a total of 8 electrode points (FP1, AF3, F3, and F7 belong to the left frontal lobe, and FP2, AF4, F4, and F8 belong to the right frontal lobe). The specific electrode point locations are shown in Figure 3. FAA (ln [right PSD]-ln [left PSD]) was used to assess the difference in the intensity of activity between the left and right frontal lobes. If FAA > 0, it means that the subject is currently in a good state and has not developed a competence frustration; if FAA < 0, it means that the subject has experienced a competence frustration.

### 3.2 Experiment on the effectiveness of interventions for competence thwarting

This study included two EEG experiments that have the same experimental materials, data acquisition and preprocessing methods as those in Experiment 1, but with a more complex experimental procedure.

#### 3.2.1 Subject

Using the G*Power tool, the minimum sample size required for a one-way two-level within-subjects ANOVA was calculated to be 34 (taking a medium effect size of *f* = 0.5, probability of making an error of *α* = 0.05, and test validity of 1 - *β* = 80%). The subjects of interest are 108 in-service firefighters and rescue personnel were recruited to participate in the experiment; all subjects met the requirements of being right-handed, having normal visual acuity or corrected vision, and having no history of neurological or psychiatric disorders; the age ranged from 23 to 37 years old, with an average age of 28.45 years old (SD = 3.45 years old); the 108 firefighters were randomly divided into three groups of 36, with one group employing the intervention of Developmental Feedback, and the second group using the intervention method of Compassion Focused Therapy, the third group was the control group and no intervention was applied. Informed consent was obtained from all subjects for this study.

#### 3.2.2 Experimental procedure

The experiment was divided into four phases, and the first two stages were the same as Experiment 1, including resting for 2 min with eyes closed (resting state) and stopping the stopwatch task to trigger the competence need frustration. Considering that firefighters were more responsive than the students in the previous experiment, the task success interval in this stage was set to [2.95 s ~ 3.05 s], so that the firefighters could be better triggered to frustrate the competence need. In the third stage, different groups will take different actions. Among them, the specific operation of the developmental feedback intervention method tells the subjects the techniques and methods of completing the stopwatch stopping task in advance to improve the success rate of the task. The specific operation of the empathy-focused therapy intervention method is to help the individual develop and cultivate a warm, safe, and soothing inner experience by making the subject feel empathy and self-compassion through verbal guidance, so as to reduce the individual’s frustration of competency needs. The specific words were such as “You have done a good job; do not be too harsh on yourself.” “Give yourself some encouragement and affirmation, no matter what you do, you are the best version of yourself,” “Believe in yourself, you are capable of doing better!” etc. The control group received no intervention. In the fourth stage, the subjects were required to complete the stopwatch task again to test the effects of the developmental feedback and empathy-focused therapy intervention method. In order to reduce the impact of subjects’ proficiency in performing the same experiment twice, the success interval of the task in this stage was set as [2.96 s ~ 3.04 s].

#### 3.2.3 Data processing

The processing of EEG data is equally capable of competence frustration induction experiments, with the difference that it was divided into three segments. The first two segments are the same as in Experiment 1, and the third segment is the EEG data from the second stopwatch task.

## 4 Results and analysis

### 4.1 Experimental results of competence need frustration induction

The EEG results of the subject’s level of competence frustration are shown in Table 1, in the resting state, the subject’s left frontal PSD value is smaller than the right frontal PSD value, and its FAA = 0.082 > 0, indicating that the subject did not suffer from competence need frustration at this time; after the stopwatch stopping experiments, its FAA = −0.081 < 0, indicating that the subject suffered from competence need frustration at this time and frontal *α*-asymmetry can reflect the competence need frustration level of the subject, proving the effectiveness of FAA. The experimental results were analyzed using a paired t-test, which showed a significant main effect of game-induced competence frustration, t (35) =3.716, *p* = 0.004, Cohen’ d = 0.94.

**Table 1:** Power spectral density values and frontal *α*-asymmetry indices (M ± SD).

Indicators	Resting state(M ± SD)	Frustrated state(M ± SD)
Left PSD (μV2/Hz)	28.78 ± 7.46	32.82 ± 6.54
Right PSD (μV2/Hz)	31,23 ± 8.80	30.27 ± 4.78
FAA	0.082	-0.081

The results of the Competence Need Frustration Scale are shown in Table 2, with Cronbach ‘s α of the questionnaire = 0.756 (α > 0.7), KMO = 0.801 (>0.5), *p* < 0.01; after completing the stopwatch stopping experiment, the mean value of the subjects’ Competence Need Frustration was *M* = 4.51 (SD = 1.52), and M > 4, indicating that the subjects also have experienced competence need frustration in their subjective cognition, which also verified the experimental results of EEG data.

**Table 2:** Results of subjective ratings of subjects’ competence needs frustration scale.

Title	Mean	Standard deviation	Cronbach ‘s α	KMO	*P*
1. I do not think I can finish the stopwatch stopping time game better	4.21	1.53	0.756	0.801	<0.001
2. I was disappointed with my performance in the stopwatch stopping time mini-game	4.61	1.93			
3. I lack confidence in my ability to do well in the stopwatch stopping time mini-game	4.45	1.25			
4. I felt like a failure when completing the stopwatch stop time game because I made so many mistakes	4.94	1.21			

### 4.2 Experimental results of competence need frustration intervention

The results of the EEG experiments of the fire rescue personnel are shown in Table 3. After conducting the first stopwatch stopping experiment, three groups of fire rescue personnel entered into the Competence Needs Frustration state, but different intervention methods achieved different intervention effects. The results for the control group demonstrated that they were in the competence need frustration state on both stopwatch stopping tasks, and their frustration levels appeared to decline to a certain extent on the second task, in the absence of external intervention. After the developmental feedback intervention method, the fire rescue personnel improved their task correctness from 22.1 to 31.8% compared to the control group’s correctness from 22.3 to 25.7%, which was a significant improvement, but FAA score became smaller instead, indicating that the developmental feedback method did not reduce their frustration, although it did improve their competence. Under the intervention of sympathetic focus therapy, the value of FAA of the subjects’ competence need frustration level changed from −0.066 to 0.011, indicating that the intervention method of sympathetic focus therapy achieved its effect and reduced their competence need frustration level. Paired *t*-test of the two groups before and after frustrations showed a significant main effect of the developmental feedback intervention method, t (36) =2.229, *p* < 0.001, Cohen’s d = 0.73, and a significant main effect of the compassion -focused therapy intervention method, t (36) = −2.687, *p* < 0.001, Cohen’s d = 0.76.

**Table 3:** Results of intervention experiments of competence requires frustration (M ± SD).

Intervention methods	Indicators	Resting state	Frustrated state	Second frustrated state
The control group	Left PSD(μV2/Hz)	36.72 ± 12.24	43.91 ± 16.13	45.23 ± 15.95
	Right PSD(μV2/Hz)	39.89 ± 13.84	41.11 ± 11.84	43.35 ± 13.08
	FAA	0.083	−0.066	−0.042
Developmental feedback	Left PSD(μV2/Hz)	43.77 ± 13.01	43.25 ± 9.73	49.62 ± 10.44
	Right PSD(μV2/Hz)	46.97 ± 15.03	41.29 ± 7.09	43.36 ± 8.20
	FAA	0.070	−0.050	−0.135
Compassion-focused therapy	Left PSD(μV2/Hz)	47.21 ± 13.53	43.47 ± 13.56	43.70 ± 14.22
	Right PSD(μV2/Hz)	49.34 ± 13.91	40.96 ± 9.54	46.50 ± 14.31
	FAA	0.044	−0.066	0.062

**Figure 4:**
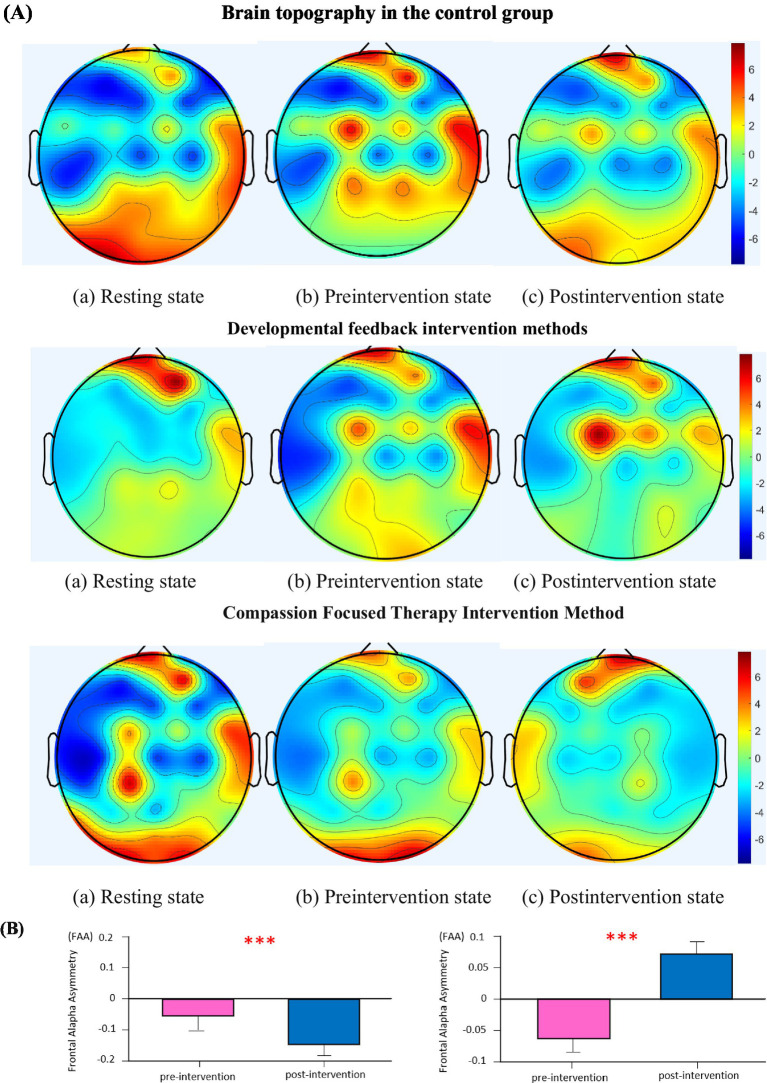
Brain topography of fire rescue personnel and FAA Bar chart. **(A)** Demonstration of the effects of brain topographic maps of the two intervention methods. **(B)** Changes in FAA before and after intervention (*p* < 0.001).

In the field of neurophysiology, alpha wave (8 ~ 13hz) can be used to respond to the information of emotional processing. Among alpha waves, 10hz is the most typical frequency, and it is also one of the most commonly used frequencies for plotting brain topography in Matlab. This study uses 10hz as an example to draw and analyze the frequency domain topographic maps of fire rescue personnel in the resting state, frustration state, and second frustration state during the experiment. Different firefighters and rescue personnel have consistent brain area activities in the same experimental task, and the brain topographic map of one of the firefighters is shown in Figure 4, where the different shades of color in different areas represent the EEG power levels of the brain regions.

## 5 Discussion

### 5.1 Developmental feedback intervention outcome discussion

In the present study, the results of the control group demonstrated a reduction in their level of competence need frustration, despite the elevated difficulty of the second stopwatch stopping task. This outcome may be attributed to the gradual familiar with the game as time elapsed, which may have led to the subconscious acceptance of their level of competence. In contrast, the primary effect of the developmental feedback intervention approach was statistically significant, with an increase in the level of competence frustration, as indicated by the EEG results. The phenomenon can be more accurately explained by self-efficacy theory. Self-Efficacy refers to the level of confidence people have in their ability to perform specific work behavior using the skills they possess, which is related to a specific domain. The higher the self-efficacy, the more likely they are to excel in a particular area of work and the more motivated they are to do it (Wang, 2024). The main factors influencing the formation of self-efficacy are the experience of success or failure of one’s own behavior, alternative experience or imitation, verbal persuasion, emotional arousal, and situational conditions. When subjects from fire rescue personnel undergo developmental feedback interventions, they focus their attention more on skills and task completion; After mastering experimental skills, their self-efficacy increases, and they will have higher expectations of their own ability and correctness, but with it comes greater task stress. Therefore, their correctness rate subsequently increased in the stopwatch stopping time task, but the failed rounds triggered more intense frustration, leading to an increase instead of a decrease in the level of competency need frustration. It is important to acknowledge that this is the immediate outcome of the intervention. The long-term impact of the intervention remains to be observed.

### 5.2 Compassion focused therapy intervention outcome discussion

A comparison of the results with those of the control group revealed that the sympathetic focusing therapy intervention was effective in freeing the subjects from the competence need frustration state. The main effect of the intervention was significant, and the results of the EEGs also demonstrated a significant change in the PSD power of the right and left frontal lobes in the different states. The specific reasons can be explained by Evolutionary and neuroscientific models of emotion regulation. Tt suggests that individuals have three main emotion regulation systems, namely the threat and self-defense system, the drive and reward system, and the affinity or comfort system. When the regulatory systems are imbalanced, especially when the threat system is not regulated, a number of mental health problems will occur (Gilbert, 2014). In the experiment, the empathy-focused therapy required subjects to focus more on themselves, allowing themselves to feel social connection and comfort from others or themselves, and balanced the relationship among the three by stimulating the comfort system, reducing both the task stress on fire rescue personnel and their levels of competence frustration. Furthermore, it was observed that the application of compassion-focused therapy did not result in an enhancement of competence. Instead, it was found to have the effect of reducing the level of competence frustration, through the provision of psychological reassurance. Managers should consider whether to adopt this intervention that lowered competence-need frustration while decreasing actual competency may not be desirable.

In fact, there are many reasons why fire rescue personnel suffer from competence need frustration. Managers should develop appropriate intervention methods based on the actual specific situation and different triggers of competence need frustration to reduce the negative effects of competence need frustration.

### 5.3 Research implications and prospects

This paper adopts the method of EEG experiment to explore the intervention measures for fire rescue personnel who suffered from competence need frustration, expands the research content of competence need frustration, and promotes the development of neuromanagement as an emerging discipline. At the same time, managers pay more attention to fire rescue personnel’s need for competence and help the construction of a mental health system for fire rescue personnel.

This study has the following limitations. First, this study examined the effects of two intervention methods in a laboratory scenario, and in the future, VR technology and other simulation methods can be used to simulate the real work scenarios of fire rescue personnel to improve the reliability of the data. Second, there are differences between the laboratory scenarios and the real-life situations, and it is necessary to study the effectiveness of the two intervention methods for competency need frustration in real-life management. Third, this study did not consider the influence of many factors such as subjects’ personality, rescue experience, etc., and the influencing factors of the effectiveness of competency-defeating intervention can be further explored in the future.

## 6 Conclusion

(1) Alpha asymmetry in the frontal lobe can be used as an EEG indicator for judging the level of competence frustration; (2) The difference in the effects of the two intervention methods indicates that when intervening in competence frustration, it is more likely to be reduced by focusing on the subjects themselves than by focusing on the completion of the task.

## Data Availability

The raw data supporting the conclusions of this article will be made available by the authors, without undue reservation.
